# The importance of genetic testing for dystonia patients and translational research

**DOI:** 10.1007/s00702-021-02329-9

**Published:** 2021-04-19

**Authors:** Jelena Pozojevic, Christian Beetz, Ana Westenberger

**Affiliations:** 1 Institute of Neurogenetics, University of Lübeck, Ratzeburger Allee 160, BMF, Building 67, 23538 Lübeck, Germany; 2 CENTOGENE GmbH, Rostock, Germany

**Keywords:** Genetic testing, Dystonia, Precision medicine, Genetic modifiers

## Abstract

Genetic testing through a variety of methods is a fundamental but underutilized approach for establishing the precise genetic diagnosis in patients with heritable forms of dystonia. Our knowledge of numerous dystonia-related genes, variants that they may contain, associated clinical presentations, and molecular disease mechanism may have significant translational potential for patients with genetically confirmed dystonia or their family members. Importantly, genetic testing permits the assembly of patient cohorts pertinent for dystonia-related research and developing therapeutics. Here we review the genetic testing approaches relevant to dystonia patients, and summarize and illustrate the multifold benefits of establishing an accurate molecular diagnosis for patients imminently or for translational research in the long run.

## Introduction

The term dystonia refers to a large group of etiologically heterogeneous disorders, some of which are undoubtedly genetically determined. The latter is obvious not only from numerous dystonia families described to date but also from a multitude of currently known genes, the pathogenic variants in which elicit dystonic symptoms. Indeed, since the discovery of the first dystonia gene almost 30 years ago (Ichinose et al. 1994), over 40 genes have been related to isolated, combined, and complex hereditary dystonia forms (Marras et al. 2016). This recognition of causative genetic defects allowed for delineating clinical phenotypes and prognoses and gaining insights into molecular mechanisms and potential therapies (Lohmann and Klein 2013; Gonzalez-Latapi et al. 2021). When put in practice through genetic testing as an initial and critical step, this amassed medical, genetic, and functional evidence may have an immediate and major impact on patients and their families. Furthermore, genetic diagnoses permit the assembly of patient cohorts pertinent to studying multiple aspects of a particular hereditary dystonia form. Despite its clear advantages, genetic testing is still conducted infrequently in the dystonia diagnostic procedure and is far from being a routine initial step (Gatto et al. 2021; Mohammad et al. 2019). The present review aims at summarizing the manifold arguments in favor of such an extended diagnostic workflow for dystonia patients.

## Genetic testing in dystonia

### Testing methods

Genetic testing refers to examining a person’s DNA sequence or chromosome structure to identify relevant changes. Methodologically, types of genetic tests that dystonia patients may undergo range from relatively simple single-variant testing and single-gene Sanger sequencing to advanced next-generation sequencing (NGS)-based approaches such as NGS gene panels, clinical exome sequencing (CES), whole-exome sequencing (WES), or whole-genome sequencing (WGS). In some instances, copy number variation (CNV) analysis may also be required. Which approach or technology will be used for genetic testing should be decided on a patient-by-patient basis as it relies on the clinical presentation at onset and at the latest examination, family history, availability of the specific diagnostic test, and the expertise in neurogenetics and experience of the physician.

*Single-variant testing* can be applied for diagnostic genetic testing in hereditary dystonia forms in which all patients are expected to carry the same variant. This is frequently due to the *founder effect*, i.e., the occurrence of a novel pathogenic variant in an early member (ancestor) of the specific patient population. At least two different genetic dystonia forms are currently known to be caused solely or primarily by such single founder variants: X-linked dystonia–parkinsonism (XDP; DYT/PARK-*TAF1*) (Lee et al. 1976) and *TOR1A*-variant related isolated dystonia (DYT-*TOR1A*) (Ozelius et al. 1997). XDP has been reported exclusively in patients of Filipino origin who manifest with adult-onset dystonia–parkinsonism and who all carry the founder ~ 2.6 kb SVA (short interspersed nuclear element, variable number of tandem repeats, and Alu composite) retrotransposon insertion in the *TAF1* gene (Makino et al. 2007; Domingo et al. 2015; Rakovic et al. 2018; Aneichyk et al. 2018). Thus, in an adult patient originating from the Philippines and with dystonic symptoms typically in the craniocervical region and/or the limbs, that may be accompanied by parkinsonian symptoms characterized by axial rigidity, bradykinesia, and postural instability (Weissbach et al. 2020), analysis of the presence of the SVA insertion is a reasonable first-line genetic testing option. This single-variant testing is performed via long-range polymerase chain reaction (PCR)-based amplification of the relevant *TAF1* region and subsequent gel electrophoresis (Kawarai et al. 2013). Similarly, DYT-*TOR1A* is a form of early-onset isolated dystonia most frequently elicited by an in-frame deletion of three nucleotides (c.907_909delGAG) in the fifth exon of the *TOR1A* gene (Ozelius et al. 1997). This variant is estimated to account for approximately 80–90% of early-onset dystonia in the Ashkenazi Jewish population (Ozelius and Bressman 2011), where it arose in a founder ~ 350 years ago (Bressman et al. 1994; Risch et al. 1995). Nevertheless, evidence shows that c.907_909delGAG has hitherto arisen de novo several times and can hence be found in patients with no Jewish ancestry (Valente et al. 1999; Ikeuchi et al. 2002). Given the high sensitivity (0.96%) and specificity (0.88%) for prediction of finding this trinucleotide deletion in Ashkenazi Jewish individuals with disease onset in limbs and before 24 years of age (Bressman et al. 2000), sequencing the *TOR1A* exon 5 is a pragmatic genetic testing approach in these patients.

*Single-gene Sanger sequencing* refers to sequencing each exon comprising the coding region of a gene of interest. This type of genetic testing has been extensively used in the past, prior to the rapid evolution and expansion of NGS-based technologies. Nowadays, the utility of the time-consuming and labor-intensive single-gene Sanger sequencing is limited to patients with dystonia phenotypes clearly pointing to a particular genetic form. For instance, in a patient with a positive family history of paternally inherited myoclonus dystonia that occurs in the first decade of life and manifests predominantly in the neck and upper limbs (Weissbach et al. 2020), the *SGCE* gene should be sequenced as this patient is likely to suffer from *SGCE*-related myoclonus dystonia (MYC/DYT-*SGCE*). Similarly, a childhood-onset foot dystonia, spreading cranially to become generalized dystonia with concomitant mild parkinsonism, diurnal fluctuations of symptoms, and excellent response to l-dopa, is highly indicative of dopa-responsive dystonia (DRD; DYT/PARK-*GCH1*) resulting from pathogenic variants in the *GCH1* gene (Weissbach et al. 2020).

*NGS-based approaches*, NGS gene panels, WES, and WGS, all rely on comparable technology and differ in the scope, depth, coverage, and cost of DNA sequencing. NGS dystonia panels are designed for sequencing the exonic regions of genes known to be associated with dystonic phenotypes, and they ensure deep and uniform coverage of the analyzed regions and, thus, high sensitivity and specificity of variant detection. Clinical exome sequencing (CES) represents a variant of WES, i.e., a sequencing analysis directed only to the coding regions of > 4000 genes reported to harbor variants hitherto associated with human genetic disorders (Trujillano et al. 2017; Gorcenco et al. 2020), rather than analyzing the exons of > 20,000 known human genes. The important limitation of the two aforementioned and otherwise most cost-effective NGS approaches is that, in patients with negative findings, they do not permit research pursuits of novel dystonia genes or reanalysis of data once novel dystonia genes have been identified.

WES and WGS provide knowledge about all exonic or genomic variants within an individual, respectively. Thus, they may detect variants in genes that are not part of the NGS panels or CES and would hence be missed by both of those less inclusive sequencing tactics. Of note, WGS is even more comprehensive than WES and may detect disease-causing variants in deep intronic or relevant regulatory regions of dystonia genes that would be overlooked by sequencing exons alone. Indeed, the merits of using WGS even as a first-tier stand-alone genetic test are being increasingly recognized (Lionel et al. 2018; Kumar et al. 2019; Bertoli-Avella et al. 2021). The conventional practice includes sequential testing that resorts to WGS only in patients who remain without a diagnosis after several other variant detection strategies have been exhausted [with reported medians of three tests and over 5000 US$ costs per patient (Lionel et al. 2018)]. Clearly, this approach leads to a significant diagnostic delay that may average 5 years and in extreme cases reach 50 years (Bertoli-Avella et al. 2021).

Several small-scale studies reporting the utility of different NGS approaches in dystonia have been published to date. In two cohorts of ~ 60 mostly early-onset, isolated, and suspected hereditary dystonia, diagnostic yields following panel sequencing of 94 and 148 genes, were 15 and 25%, respectively (van Egmond et al. 2017; Ma et al. 2018). Importantly, in a smaller (*n* = 16) but even more carefully chosen group of early-onset dystonia patients (mean age at onset of 10 years, normal brain magnetic imaging findings, and non-responsive to levodopa), WES focusing on 225 dystonia-related candidate genes identified clinically relevant variants in 37.5% of screened individuals (Zech et al. 2017). Hence, in preselected patients with a likely genetic etiology of dystonia, the probability of establishing a genetic diagnosis correlates with the number of investigated genes. Frequently, dystonia is a part of a complex neurological and sometimes multisystemic disease presentation (Herzog et al. 2020). In such cases, phenotypic clues are too diverse and unspecific, and a wide-reaching type of genetic testing is instrumental and crucial for arriving at a proper clinical diagnosis. A study performing WES in 189 dystonia patients (with over two-thirds of participants presenting with co-occurring neurological manifestations such as intellectual disability or delayed development, seizures, and psychiatric issues) reported positive genetic findings related to dystonia in 22% of participants (Powis et al. 2020). Approximately 85% of patients that received a diagnosis previously underwent at least one type of genetic testing that was inconclusive (Powis et al. 2020). The only study to date describing WGS diagnostic yield and findings in dystonia patients also investigated probands with heterogeneous disease presentation (Kumar et al. 2019). Of 111 participants, a genetic diagnosis could be made for 12% and was more likely in patients with a younger age at onset and earlier age at testing (Kumar et al. 2019). Interestingly, the probability of receiving positive genetic testing results was significantly higher in the group with a combined dystonia phenotype, whereas this probability was lower among patients with focal or segmental isolated dystonia with onset in adulthood (Kumar et al. 2019). It is noteworthy that CNVs were detected in three (23%) of the diagnosed individuals (Kumar et al. 2019).

Thus, while in patients with childhood-onset isolated dystonia and positive family history gene panel sequencing may be a cost-effective but still beneficial option, in probands affected by dystonia as a part of a more complex clinical phenotype, WES and WGS represent a more meaningful and rewarding strategy, as these phenotypes are not likely to be explained by variants in isolated dystonia genes.

*CNV analyses* include testing for the presence of CNVs. CNVs are deletions and duplications (i.e., changes in the number of copies) of large segments of DNA that may encompass single exons, groups of exons, entire genes, or even two or more adjacent genes. When choosing a test and/or analyzing the data, the potential existence of pathogenic CNVs should be considered. Namely, NGS-based genetic testing can and should also be examined for evidence of CNVs (Abyzov et al. 2011; Roller et al. 2016). In absence of NGS data, the occurrence of CNVs can be assayed by real-time quantitative PCR (qPCR), multiplex ligation-dependent probe amplification (MLPA), or digital droplet PCR (ddPCR). Of note, in contrast to qPCR and ddPCR that can inspect only one exon at a time, MLPA permits simultaneous quantification of 20–30 exons of several genes. CNV analysis is usually utilized to complement single-gene sequencing, when the latter returns a negative test result, and when there are indications that the disease phenotype is associated with changes in a particular gene. To date, CNVs were identified in four dystonia genes, namely *THAP1* (Baker et al. 2014; Brüggemann et al. 2015), *KMT2B* (Zech et al. 2016), *GCH1* (Djarmati et al. 2007), and *SGCE* (Grünewald et al. 2008). Thus, if, for instance, the patient with childhood-onset, paternally inherited myoclonus dystonia from our previous example (see “*Single-gene Sanger sequencing”*), does not harbor small sequence changes in the *SGCE* (or *KCTD17*) gene, analysis of CNVs in *SGCE* would be warranted. Large deletions within chromosome 7 encompassing *SGCE* and a few neighboring genes, one of which is *COL1A2*, have been reported in myoclonus dystonia patients (Grünewald et al. 2008). Interestingly, given that *COL1A2* encodes a component of collagen, these individuals display additional non-motor phenotypes such as delayed skeletal development, osteoporosis, and cartilage defects (Grünewald et al. 2008). Hence, in a patient with myoclonus dystonia and the aforementioned bone-related abnormalities, quantitative *SGCE* analysis is a sensible initial genetic testing approach.

### Testing utility

**Fig. 1:**
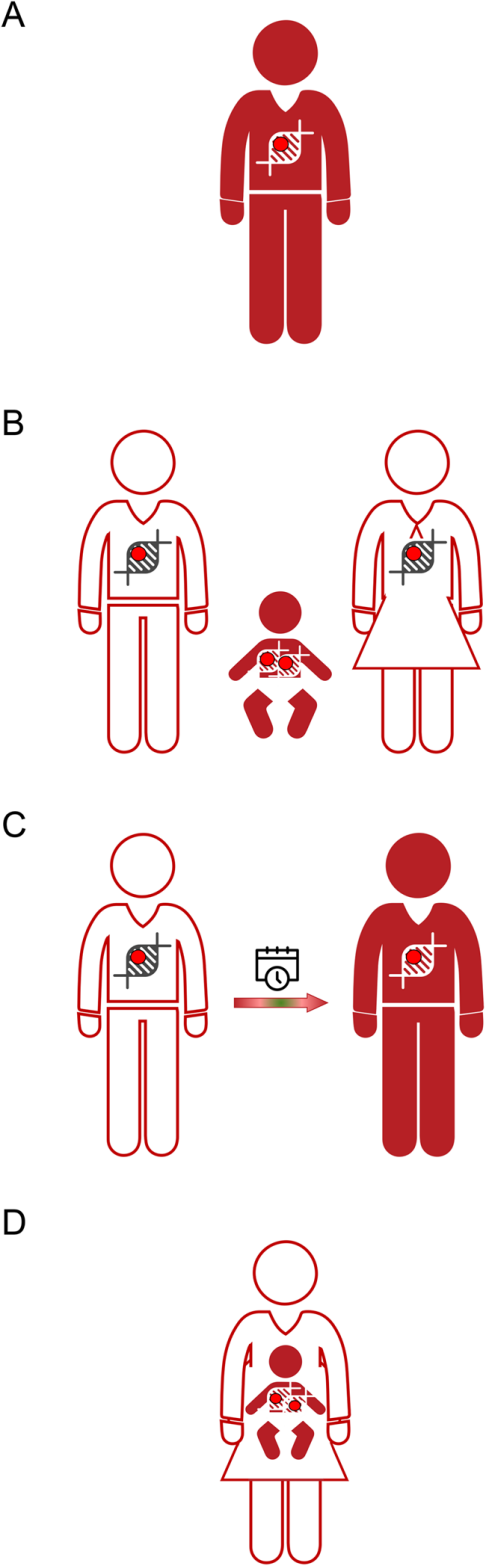
Individuals eligible for genetic testing. **a** Patients seeking genetic diagnoses should be screened by diagnostic genetic testing. **b** Individuals suspected to carry a pathogenic variant in a single copy of a gene related to autosomal recessive dystonia forms may undergo carrier genetic testing to determine their chance of having an affected offspring. **c** Clinically unaffected individuals whose family members suffer from dystonia are at risk of carrying a variant that will predispose them to developing symptoms over time (i.e., later in life) and are thus eligible for predictive/presymptomatic genetic testing. **d** Prenatal genetic testing is performed to assess the presence of a presumed disease-causing change (or changes) in a fetus prenatally. Affected individuals are shown in red and unaffected in white

Based on the individual’s medical circumstances they are designed to address, types of genetic testing pertinent to dystonia are: (1) diagnostic testing, (2) carrier testing, (3) predictive/presymptomatic testing, and (4) prenatal testing. Of these, *diagnostic genetic testing* is most commonly used, given that it may be instrumental for establishing a genetic diagnosis in affected individuals, i.e., determining which genetic form of dystonia the patient suffers from (Fig. 1a). If informative, the diagnostic genetic test will result in the identification of a DNA defect (and concomitantly the gene in which it is situated) responsible for the disease. The variety of techniques utilized for diagnostic genetic testing and the clinical contexts in which they are most effective have been discussed in the “Testing methods” section.

*Carrier genetic testing* is important for the individuals suspected to carry a pathogenic variant in a single copy of a gene related to autosomal recessive dystonia forms. Such individuals are unaffected but have a family history of dystonia and, if tested positive, have an increased chance of having an affected child (Fig. 1b).

Similar to carrier genetic testing, *predictive/presymptomatic genetic testing* is also offered to clinically unaffected individuals whose family members suffer from dystonia. However, these individuals are at risk of carrying a variant that will predispose them to developing symptoms later in life (Fig. 1c).

Finally, *prenatal genetic testing* is performed to assess the presence of a presumed disease-causing change (or changes) in a fetus before birth (Fig. 1d). In the last three applications (i.e., to investigate whether a person carries a particular DNA alteration), single-variant testing is most appropriate.

The effects of genetic testing on tested individuals and possibly on their family members are multifaceted and generally advantageous (Lohmann and Klein 2014; Lingen et al. 2016; Krabbenborg et al. 2016; Mollison et al. 2020). Upon receiving conclusive results, relief from uncertainty and reduction of psychological distress are the common denominators in terms of benefit for all aforementioned motives for genetic testing. A definitive genetic diagnosis assists communication with healthcare professionals, but also with other involved individuals or social contacts, such as teachers, employers, support groups, friends, and family members. Furthermore, it may provide an organic cause to a previously presumed psychogenic (i.e., functional) movement disorder or may preclude infliction of iatrogenic harm through ineffective and unnecessary treatments (e.g., surgery). Clarification of the genetic basis of dystonia in a patient enables making informed life decisions not only for the affected proband but possibly for his/her relatives. Importantly, in some cases, genetic diagnosis may illuminate particularly effective therapeutic and clinical management options and provide a more specific prognosis (Brüggemann and Klein 2019).

## Precision medicine and translational research in dystonia

### Precision medicine

**Fig. 2:**
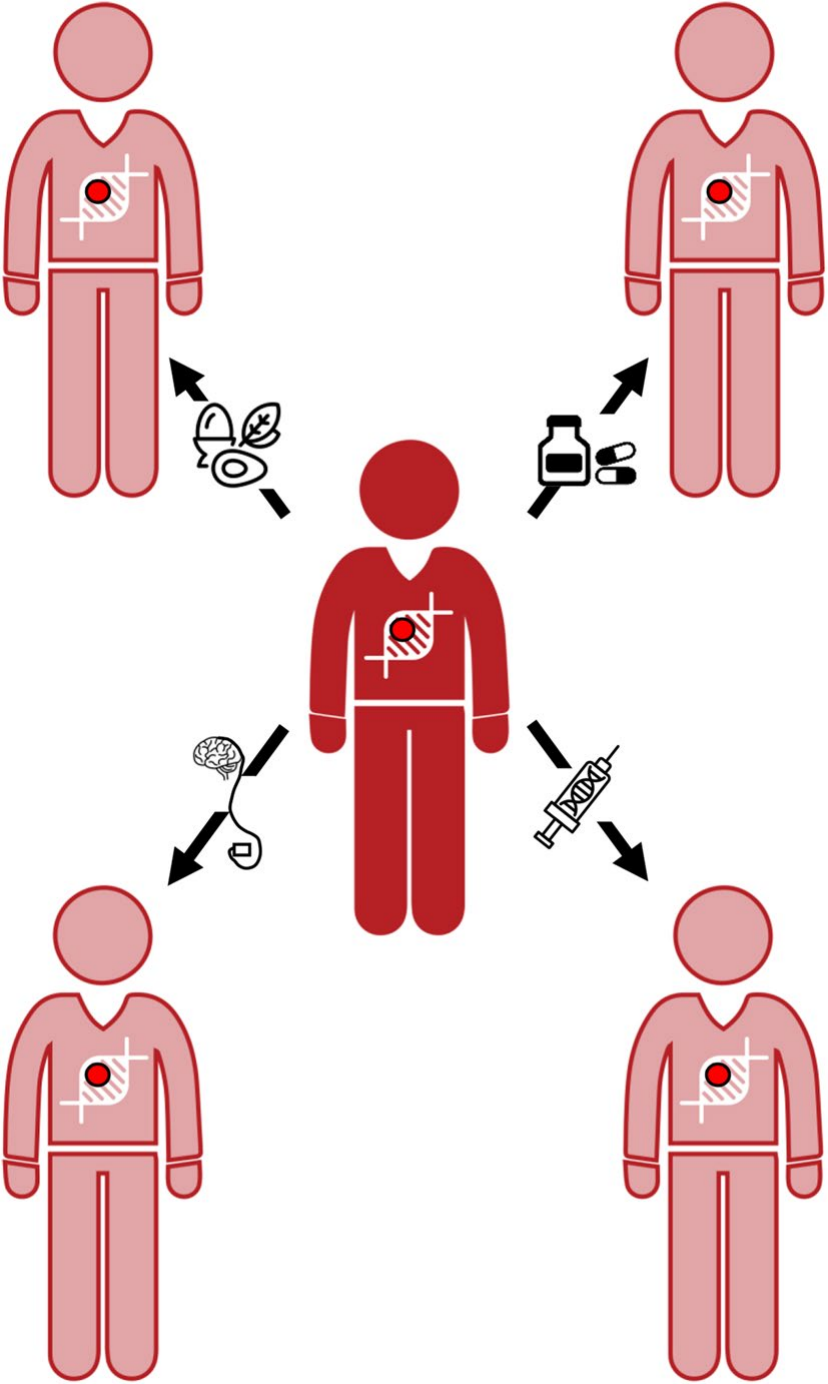
Depending on the causative gene defect, some forms of dystonia can be successfully treated and show considerable disease symptom improvements upon gene-defect-determined dietary treatment, medication, or a particular medical procedure. Affected individuals are shown in red and individuals with less severe symptoms upon treatment in light red

To define the most effective treatment approaches for a given patient, *precision medicine* integrates and considers knowledge of a spectrum of individual data (including clinical, genetic, lifestyle, and, if possible, biomarker information) in addition to the personal approach that is an intrinsic part of the patient–physician relationship (König et al. 2017). Thus, the first steps in therapeutic decision-making for a dystonia patient include recognizing the type and the extent of dystonia, identifying associated comorbidities, and defining the underlying etiology (Mohammad et al. 2019; Jinnah 2020). Different genetic types of dystonia result from defects in different genes and consequently different pathophysiological mechanisms. Therefore, the recognition of the implicated gene or even type of the pathogenic variant may significantly influence the therapeutic management of patients. Considerable disease symptom improvements that may even be life-saving, can in some instances be achieved by gene-defect-determined dietary treatment, medication, or a particular medical procedure (Fig. 2) (Jinnah et al. 2017, 2018; Brüggemann and Klein 2019; Jinnah 2020; Tisch and Kumar 2021).

For example, a heterozygous missense variant in the *SLC2A1* gene, encoding glucose transporter 1 (GLUT1) that is responsible for the entry of glucose into the brain, may cause a complex phenotype including paroxysmal dystonia due to the reduced levels of glucose in the CNS (Leen et al. 2013). A ketogenic diet, which provides an alternative energy source for brain metabolism, may improve or relieve symptoms, and patients treated at a younger age have a more favorable prognosis (Alter et al. 2015).

Dopa-responsive dystonia (DYT/PARK-*GCH1*) develops due to pathogenic variants in the *GCH1* gene, which codes for one of the enzymes in the dopamine synthesis pathway (GTP-cyclohydrolase 1). The reduction of dopamine can be effectively mended by dopaminergic medication resulting in a life-long alleviation of motor symptoms. In some cases, upon positive prenatal genetic testing, even prenatal replacement therapy with levodopa may be indicated (Brüggemann et al. 2012). The above examples further underline not only the importance of genetic testing but also the need for its timeliness.

An increasing body of evidence implies that outcomes and even surgical targets of treatment procedures such as deep brain stimulation (DBS) may be corelated with an underlying genetic cause (Brüggemann et al. 2015, 2019; Jinnah et al. 2017). Patients affected due to the c.907_909delGAG *TOR1A* variant respond well to DBS and more predictably so than the carriers of various *THAP1* pathogenic changes (Brüggemann et al. 2015). In addition, a very recent investigation of DBS effects in 18 *KMT2B* variant carriers reports significant initial improvement (Cif et al. 2020). When considering combined dystonia forms, particularly promising outcomes can be expected in XDP patients (Brüggemann et al. 2019; Abejero et al. 2019) and myoclonus dystonia patients with *SGCE* variants (Kosutzka et al. 2019), but not for *ATP1A3*-related dystonia (DYT/PARK-*ATP1A3*) (Brücke et al. 2015; Albanese et al. 2017; Tisch and Kumar 2021).

Although currently unavailable for any of the hereditary dystonia forms, genetic therapeutic approaches, that profoundly and intrinsically rely on precise molecular diagnoses, are crucial for patients for whom hitherto only symptomatic management was feasible. Namely, scientific and technological advances have already allowed the medical application of therapies that can reinstate lost gene function via viral transgenic expression (Li and Samulski 2020; Ravi et al. 2021) or alleviate the harmful effect of abnormally functioning genes by neutralizing or modulating their mRNAs through antisense oligonucleotides or RNA interference (Mercuri et al. 2018; Levin 2019; Tabrizi et al. 2019; Roberts et al. 2020). Therapeutic/curative genome editing is emerging as a powerful but extremely costly next-generation therapeutic intervention due to its inherent requirement to be tailored for individual patients (Cox et al. 2015; Wilson and Carroll 2019; Doudna 2020). This strategy represents a fundamental game-changer as it holds the potential to replace a chronic disease treatment with a “one-and-done” cure in the future (Doudna 2020).

### Translational research

Unraveling the genetic basis of dystonia in a patient or, alternatively, failing to make a molecular diagnosis due to a negative finding upon comprehensive genetic testing, both have important roles in designating the individuals who may contribute to translational research. Furthermore, even identification of (currently) unaffected individuals with disease-causing variants may provide important clues and resources for studies aiming to understand “the genetics of health” or to develop and implement therapies in the prodromal or early disease stages (Brüggemann and Klein 2019).

As already noted in the “Genetic testing in dystonia” section, WES and WGS may be utilized for the discovery of novel dystonia-relevant genes in individuals with unresolved disease etiology (i.e., negative genetic testing results) through a number of strategies (Gilissen et al. 2012). The analysis of the WGS/WES data in several unrelated patients with similar specific and distinguishing phenotypes may indicate that the majority (or at least a few) of the probands carry plausibly disease-causing variants in the same gene. In more clinically heterogenous disorders, such as dystonia, the cohorts of investigated patients should be larger. For instance, a recent study of WES data from 138 individuals with unresolved generalized dystonia detected loss-of-function variants in the *VPS16* gene in 6 probands (Steel et al. 2020). Importantly, through international collaborations and swift searches through already existing WES/WGS data of dystonia patients, 13 additional individuals with *VPS16* disease-causing variants were identified (Steel et al. 2020). Along the same lines, large and comprehensive genotype–phenotype data repositories offered and curated by diagnostic genetic testing companies are a powerful resource for identifying new disease genes or evaluating/confirming the clinical significance of the candidate genes or variants (Dulovic-Mahlow et al. 2019; Abbasi-Moheb et al. 2020; Kuipers et al. 2020; Massadeh et al. 2020). In turn, these research findings are promptly applied in diagnostic practice and sometimes fewer than 3 months since the initial report of a new disease gene are required for the given gene to become a part of commercial diagnostic gene panels (Lohmann and Klein 2014).

Genetic testing and, importantly, publication of the identified variants and detailed associated clinical, laboratory, and neuroimaging findings, as well as the patients’ responses to various therapies are instrumental for establishing and delineating genotype–phenotype correlations in dystonia. This is of particular importance for the individuals harboring pathogenic variants as it may enable a more refined prognosis for probands and understanding of genetic risk to family members. To this end, the International Parkinson and Movement Disorder Society Genetic mutation database (MDSGene, https://www.mdsgene.org/) represents an important initiative with the goal of providing a systematic and comprehensive overview of published data on movement disorder patients reported to carry disease-causative genetic variants. It currently contains information on patients harboring mutations in > 20 isolated or combined dystonia genes and several other genes that may present with dystonia. All MDSGene data are obtained and curated, utilizing a standardized data extraction protocol with predefined inclusion and exclusion criteria, from relevant scientific articles identified through PubMed literature searches. Application of this resource has already resulted in important genotype–phenotype relations for dystonia genes (Pauly et al. 2020; Lange et al. 2021).

In addition to providing a genetic diagnosis to a patient, or indicating a genetic predisposition to develop dystonia to asymptomatic persons, an important outcome of a positive genetic testing finding is the prospect for dystonia subtyping, i.e., genetic stratification of individuals for further research or clinical studies and trials.

For example, the qualifying individuals may be offered participation in studies of genetic, epigenetic, or environmental modifiers of penetrance and expressivity of a particular genetic form of dystonia. Namely, patients with pathogenic variants in the same dystonia gene frequently show wide ranges of ages at symptom onset and heterogenous severities, clinical manifestations, and disease progressions, the phenomenon referred to as variable disease expressivity. Furthermore, almost all dystonia forms have incomplete penetrance and some pathogenic variant carriers remain unaffected throughout their life (Cooper et al. 2013). Obviously, elucidating the factors contributing to health (i.e., reducing the likelihood of dystonia occurrence and diminishing the symptom severity or speed of disease progression) in mutation carriers has a high translational potential. The prerequisite for the success of such research endeavors is sizable cohorts of patients with the same main genetic etiology and available clinical and environmental data. These cohorts can be further explored by genome-wide association study (GWAS) for the presence of genetic factors modifying the given phenotype or by other statistical correlation methods to determine the role of environmental modifiers. Of note, significant genetic modifiers of (age-related) penetrance have been reported for two dystonia forms to date. Among the carriers of the c.907_909delGAG *TOR1A* variant, only ~ 30% become affected, and among patients, severity may range from generalized early-onset disease to late-onset focal dystonia. Interestingly, the frequency of a common coding polymorphism in *TOR1A*, p.Asp216His, was found to be increased in unaffected carriers and decreased in carriers with dystonia, and the penetrance associated with carrying this protective allele was further reduced to ~ 3% (Kock et al. 2006; Risch et al. 2007). Moreover, in XDP patients, who all harbor the same founder insertion, the number of repeats in the unstable hexanucleotide repeat polymorphism within the insertion has been shown to inversely correlate with age at onset (Bragg et al. 2017; Westenberger et al. 2019) and disease severity (Westenberger et al. 2019). Nevertheless, this modifier seems to account for only 50% of the variability in age at onset, implying that there are other genetic factors at play.

Imprinting represents an important epigenetic mechanism responsible for reduced penetrance in *SGCE*-related myoclonus dystonia (Müller et al. 2002). The individuals that inherit a pathogenic *SGCE* variant from their mothers will remain unaffected, as the maternal *SGCE* allele is always methylated and thus not expressed regardless of whether it is mutated or not. This phenomenon has a striking prognostic consequence for family planning in particular. To date, no environmental modifiers of dystonia have been described. However, the recently reported positive effect of the use of tobacco and consumption of black tea, reducing the age at onset in Parkinson’s disease patients carrying the LRRK2 p.Gly2019Ser mutation by a median of 8 and 6 years, respectively, may be used to illustrate the importance of environmental cues (Lüth et al. 2020).

The existence of reliable clinical and molecular biomarkers is a prerequisite for a precise assessment and comparison of the current state of disease among the patients (including presymptomatic and early disease stages) as well as for the prediction of dystonia outcome and monitoring of disease progression or response to treatment. Ideally, biomarkers should be developed in well-defined disease subgroups (e.g., among carriers of the same mutation), in contrast to the heterogeneous patient populations that are typically recruited into clinical studies (Espay et al. 2017).

Finally, dystonia patients (or non-manifesting carriers) selected for positive findings in a certain dystonia gene may be timely enrolled into gene-tailored clinical trials of potential disease-modifying therapies, underscoring the necessity of systematic testing in dystonia.

## Concluding remarks

Genetic testing is the only way to establish a genetic diagnosis in dystonia patients. Which approach or technology will be used depends on the availability and purpose of the genetic test (e.g., diagnostic vs. carrier screening), clinical presentation (e.g., isolated vs. combined dystonia), and the experience of the physician. A positive genetic test is crucial for appropriate genetic counseling and has the potential to improve or resolve at least some of the worrisome disease aspects for patients and their caregivers. In particularly fortunate cases, it may even indicate a life-changing treatment option. Elucidating the genetic basis of dystonia in patients also indicates the individuals who may contribute to translational research aiming to identify disease modifiers or biomarkers or participate in gene-tailored clinical trials of potential disease-modifying therapies.
